# Effect of voluntary alcohol consumption on *Maoa* expression in the mesocorticolimbic brain of adult male rats previously exposed to prolonged maternal separation

**DOI:** 10.1038/tp.2015.186

**Published:** 2015-12-08

**Authors:** M Bendre, E Comasco, I Nylander, K W Nilsson

**Affiliations:** 1 Department of Neuroscience, Uppsala University, Uppsala, Sweden; 2 Department of Pharmaceutical Biosciences, Uppsala University, Uppsala, Sweden; 3 Centre for Clinical Research, Uppsala University, County Hospital, Västerås, Sweden

## Abstract

Discordant associations between monoamine oxidase A (*MAOA*) genotype and high alcohol drinking have been reported in human and non-human primates. Environmental influences likely moderate genetic susceptibility. The biological basis for this interplay remains elusive, and inconsistencies call for translational studies in which conditions can be controlled and brain tissue is accessible. The present study investigated whether early life stress and subsequent adult episodic alcohol consumption affect *Maoa* expression in stress- and reward-related brain regions in the rat. Outbred Wistar rats were exposed to rearing conditions associated with stress (prolonged maternal separation) or no stress during early life, and given free choice between alcohol and/or water in adulthood. Transcript levels of *Maoa* were assessed in the ventral tegmental area, nucleus accumbens (NAc), medial prefrontal cortex, cingulate cortex, amygdala and dorsal striatum (DS). Blood was collected to assess corticosterone levels. After alcohol consumption, lower blood corticosterone and *Maoa* expression in the NAc and DS were found in rats exposed to early life stress compared with control rats. An interaction between early life stress and voluntary alcohol intake was found in the NAc. Alcohol intake before death correlated negatively with *Maoa* expression in DS in high alcohol-drinking rats exposed to early life stress. *Maoa* expression is sensitive to adulthood voluntary alcohol consumption in the presence of early life stress in outbred rats. These findings add knowledge of the molecular basis of the previously reported associations between early life stress, *MAOA* and susceptibility to alcohol misuse.

## Introduction

Globally, alcohol misuse is a major problem with health, economic and social consequences.^1^ Alcohol is frequently consumed for its pleasurable and euphoric effects, that is, positive reinforcing effects, but also for its sedative and stress relieving effects, that is, negative reinforcing effects.^2, 3^ The progression from controlled drinking to compulsive drinking is crucial for the development of alcohol use disorder (AUD) and involves several neuronal circuits.^3^ Excessive alcohol consumption induces transcriptional changes within different mesocorticolimbic brain regions participating in stress and reward regulation, alcohol intoxication, reinforcement and addiction.^4, 5, 6^ The reinforcing effects of alcohol (both positive and negative) are mediated by dopaminergic neurons that originates in the ventral tegmental area (VTA) and projects to the nucleus accumbens (NAc) and prefrontal cortex,^7^ as well as to the cingulate cortex (CCx), dorsal striatum (DS) and amygdala (Amg),^8^ altogether forming the mesocorticolimbic pathway. In addition, a regulatory role is played by serotonergic neurons in this pathway in mediating the rewarding and reinforcing effects of alcohol,^9, 10^ as well as by noradrenergic neurons in mediating the effects of negative emotions that arises due to withdrawal of alcohol and stress-related relapse.^11^ Monoaminergic neurotransmitters thus play a key role in mediating both the acute and chronic effects of alcohol. A meta-analysis, assessing alcohol-induced acute effects in rats on the neurochemistry of forebrain regions involved in neuro-circuits of AUD, reported an overall enhancement of monoaminergic transmission.^12^ In contrast, chronic alcohol consumption seems to have varying and region specific effects on dopaminergic neurotransmission,^13, 14, 15, 16^ while serotonergic^9, 10, 16^ and noradrenergic central neurotransmission is decreased.^16^ Moreover, long-term alcohol exposure induces changes in expression of monoaminergic genes in mesocorticolimbic brain regions of rats.^17, 18^

Functional variations in key brain circuits involved in reward are likely to influence an individual's vulnerability to develop AUD.^19^ Individual differences in the transition into compulsive drinking and proneness to AUD depend on genetic heterogeneity and polygenicity, but also on gene by environment interactions.^20^ Clinical and pre-clinical studies indicate that early life stress is a risk factor for AUD later in life.^21, 22^ One's early experience, particularly during early life, such as childhood maltreatment in humans and maternal separation (MS) in rodents, can influence the expression of genes involved in neurotransmitter systems that regulate stress response and behavior,^23^ and result in dysfunction of mesocorticolimbic brain pathways.^24, 25, 26^ This in turn can lead to stress-related disorders^27^ and alcohol-seeking behavior.^28^ However ELS does not contribute to the development of AUD alone, but in an interaction with genetic factors.^29, 30^

Growing evidence suggests that the monoamine oxidase A (*MAOA)* gene, which codes for MAOA, plays an important role in regulating behavior and is linked with impulsivity, stress-related disorders and AUD.^31^ Monoamine neurotransmitters such as serotonin, dopamine and norepinephrine, that are mediating the effects of alcohol and are crucial for reward and initiation of alcohol reinforcement,^7^ are metabolized by *MAOA* in humans^31^ as well as in rats (*Maoa*).^32^ In 1993, mutations in the *MAOA* gene, resulting in deficiency of MAOA, were identified in human males presenting abnormal behavior such as impulsive aggression, arson, and attempted rape.^33^ Later, a functional polymorphism was discovered in the promoter region of the *MAOA* gene (*MAOA*-uVNTR) in humans and shown to affect the transcriptional activity in transfected non-neuronal cells, with the 3.5 or 4-repeat allele having 2–10 times higher activity than the 3 or 5-repeat allele.^34, 35^ Whereas, *in vivo* studies do not find any association between MAOA-uVNTR genotype and its transcriptional activity, in human^36, 37^ as well as non-human primates.^38^ Several studies reported an association of both the high and low activity *MAOA*-uVNTR alleles with antisocial behavior,^39, 40, 41^ and susceptibility to AUD,^42, 43^ while others found an association with alcohol misuse but only in an interaction with stressful life events.^44, 45, 46, 47^ On the contrary, some other studies found no association between *MAOA*-uVNTR and AUD.^48, 49^ Furthermore studies of primates provide a somewhat discordant evidence. Mother-reared male rhesus monkeys carrying the low activity rh*MAOA*-uVNTR allele display higher aggression scores as adults,^50^ whereas no effect was found in adolescent and young adult males in one study,^38^ while in another study the ones carrying the high activity rh*MAOA*-uVNTR allele consumed higher amount of alcohol than those carrying the low activity allele.^51^ Altogether, these studies suggest *MAOA* as a candidate gene sensitive to gene by environment interactions and associated with AUD. However, they also call for translational studies to clarify the inconsistencies in *MAOA*-by-environment interaction effects^52, 53, 54^ and deepen knowledge on potential molecular mechanisms behind this interaction allowing accessibility of the brain. In fact, though several studies have attempted to investigate *MAOA* genotype by environment interaction and its effect on behavior and AUD, the connecting link, that is the biological basis behind such interaction, is missing. Hence, the hypothesis that early life stress and alcohol consumption, alone or in combination, would be associated with altered expression of *Maoa* in brain areas related to stress-regulation and reward processing in adult rats was tested in the present study.

The aim was to assess whether early life stress in the form of prolonged MS, and adult episodic voluntary alcohol consumption, affect *Maoa* expression in brain regions related to stress and reward in outbred rats. First, the effects of voluntary alcohol drinking and the potential confounder of single housing were investigated in rats subjected to conventional animal facility rearing conditions. Second, the long-term effect of early life stress was examined using a rodent model where rats were exposed to prolonged and brief, respectively, MS during the first three post-natal weeks^55, 56^ and to voluntary episodic alcohol drinking in adulthood. Finally, the interaction and main effect of prolonged MS and alcohol intake were investigated. Correlations between *Maoa* expression in different regions were tested in each experimental group, as well as between *Maoa* and alcohol intake in the experimental groups exposed to alcohol.

## Materials and methods

### Animals

Time-mated Wistar dams (*n*= 25; RccHan:WI, Harlan Laboratories, Horst, The Netherlands) arrived at gestation day 15. After birth (post-natal day (PND) 0), the pups were sexed and cross-fostered to avoid the use of biological littermates in the same experimental groups. Each litter was arranged to 10 pups, 6 males and 4 females, and the litters were randomly assigned to the different experimental groups. Only males were used in the present study. The study was performed all in one, the researcher was not blind to the animal experiment, however, genetic and hormone analyses were performed in a blind manner. The study was approved by the Uppsala Animal Ethical Committee (C32/11) and followed the guidelines of the Swedish Legislation on Animal Experimentation (Animal Welfare Act SFS1998:56) and the European Communities Council Directive (86/609/EEC).

### Early life rearing conditions

**Figure 1:**
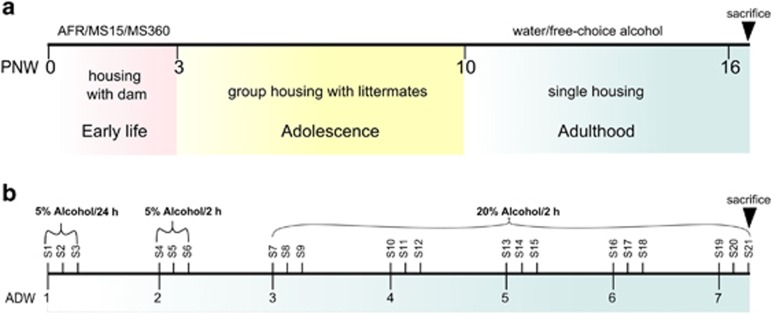
Experimental design from post-natal weeks (PNW) 0 to 16. (**a**) Male Wistar rats were subjected to MS15, MS360 or AFR the first three post-natal weeks and exposed to voluntary alcohol consumption from PNW 10 to 16. All animals, except one group of AFR rats (*n*=9), were single-housed from PNW 10. (**b**) Detailed scheme of the voluntary drinking paradigm. A two-bottle choice paradigm was used with free access to either alcohol/water or water/water for 3 consecutive days per week from PNW 10 to 16. The access to alcohol was limited to 2-h sessions. The rats were killed during ADW 7, immediately after a 2-h drinking session. ADW, alcohol-drinking week; AFR, animal facility reared; MS15, maternal separation for 15 min, MS360, maternal separation for 360 min; PNW: post-natal week; S, session.

A schematic outline of the experimental design in the study is illustrated in Figure 1. A rodent MS model was used to simulate different early life conditions during the first 3 post-natal weeks. Based on previous studies, prolonged daily MS (360 min; MS360) was used to simulate a risk environment^56^ and short MS (15 min; MS15) was used as a control to MS360. The separations were performed during the light period and started at 0900 hours. The MS procedure has been described in detail elsewhere.^57^ The litters were weighed on PND 0, 3, 7, 10, 13, 16 and the cages were changed on PND 7 and 16. The separations were always performed in the same animal rooms and only one person performed all separation and care taking. Animal facility reared (AFR) rats were included in the study for assessment of single housing and alcohol drinking in rats subjected to conventional laboratory rearing conditions. The animals in the AFR group were left undisturbed with the exception of cage change (PND 7, 16) and weighing of the litter (PND 0, 7, 16). On PND 22, all animals were weaned and the light/dark cycle was switched to lights off between 0600 to 1800 hours until the end of the experiment. The rats were group-housed, three per cage, during adolescence (Figure 1a).

### Voluntary alcohol consumption

On post-natal week (PNW) 10 the MS rats were randomly assigned to water-drinking (MS15W, *n*=10; MS360W, *n*=10) and alcohol-drinking groups (MS15A, *n*=10; MS360A, *n*=20). More rats were included in the MS360 group based on previous findings of subgroups with responder and non-responder rats regarding the MS effect on alcohol intake.^56^ The AFR rats were also assigned to water (AFRW, *n*=9) and alcohol drinking (AFRA, *n*=11), respectively. All rats were single-housed for individual fluid measurements until decapitation at PNW 16. One additional group of AFR rats (*n*=7) was group housed during PNW 10 to 16. The rats had free choice between non-sweetened alcohol (5 or 20% made from ethanol 96% Solveco, Rosersberg, Sweden) and water during the dark cycle. Water-drinking controls had two bottles with water.

A detailed scheme of the voluntary alcohol exposure paradigm is shown in Figure 1b. During the first alcohol-drinking week (ADW), the rats had free access to 5% alcohol for 24 h and the next week limited access to 5% for 2 h for 3 consecutive days a week; the following 5 weeks they had access to 20% alcohol in 2-h sessions for 3 consecutive days a week followed by 4 days of no alcohol with free access to water (Figure 1b). Alcohol and water were changed every session and the bottle position was altered every day to avoid position preference. Bottles with nipples were used to avoid spillage biases and at the end of each session the alcohol and water intake was quantified by weighing the bottles. The individual drinking patterns over time were assessed from ADW 1 to 7. At post-natal week 16, that is, ADW 7, all rats were killed. The animals were brought to a separate room for decapitation immediately after the drinking session 21 (Figure 1b). To assess correlations between individual drinking behavior and *Maoa* expression in different brain regions, the average alcohol intake during ADW 6 and ADW 7 were considered; ADW 6 because it is the drinking last week without any putative disturbances due to persons entering the room and more representative of the individual drinking pattern; and ADW 7 because it reflects the effect of most recent alcohol intake at the time-point of death which is more relevant in terms of alcohol-induced effects on gene expression. The VTA, NAc, medial prefrontal cortex (mPFC), CCx, Amg and DS were removed from the brain and immediately frozen on dry ice and stored at −80 °C. At the same time, trunk blood was collected and stored at −80 °C for studying blood corticosterone levels.

### Gene expression analyses

#### RNA isolation

RNA was isolated from rat VTA, NAc, mPFC, CCx, Amg and DS using AllPrep DNA/RNA/miRNA Universal Kit according to the manufacturer's protocol (Qiagen, Sollentuna, Sweden). Quantification of the nucleic acid was carried out using a Nanodrop ND 1000 spectrometer (Thermo Fisher Scientific, Wilmington, DE, USA).

#### cDNA synthesis

RNA (700ng) was converted to complementary DNA (cDNA) using the QuantiTect Reverse Transcription Kit (Qiagen). The manufacturer's protocol was followed including a genomic DNA wipe-out reaction. The final cDNA synthesis reaction was performed at 42 ^o^C for 35 min, and the reaction was inactivated at 95 ^o^C for 5 min. The newly synthesized cDNA was diluted 20 times with distilled water and stored at −20 ^o^C until further analysis.

#### qPCR analyses

Diluted cDNA (20 × ) was used to assess the expression of *Maoa*, as well as *Actb, Gapdh* and *Rpl19,* as housekeeping genes, using CFX96 Touch Real-Time PCR Detection System real-time PCR (Applied Biosystems, Foster City, CA, USA). Primers were designed using Primer 3 (http://frodo.wi.mit.edu/) and cross-checked using Primer Map (http://www.bioinformatics.org/sms2/primer_map.html) (Supplementary Table S1). The final reaction mixture of 20 μl contained iQ SYBR Green Supermix (Bio-Rad Laboratories, Hercules, CA, USA), 0.15μM of each primer and 3 μl cDNA template; and each sample was run in triplicates. The PCR cycling conditions were as follows: hot start at 95 ^o^C for 3 min; denaturation at 95 ^o^C for 10 s; annealing for 30 s and extension at 72 ^o^C for 45 s (Supplementary Table S1). Cycle was repeated 40 times. A three-step control was performed to assess genomic DNA contamination: (1) On column DNase treatment during the extraction process; (2) genomic DNA wipe-out reaction prior to cDNA synthesis; (3) Designing of primers across two adjacent exons to avoid any unspecific amplification of genomic DNA. Moreover, each real-time PCR plate contained samples belonging to all experimental groups. Each plate contained a positive and a negative control sample. Gel electrophoresis images illustrating the primer specificity of the genes-of-interest and the RNA integrity of 10% of randomly selected samples from the regions-of-interest are presented in the Supplementary Figure S1.

#### Data analysis

Data of the relative fluorescence unit were collected using Biorad CFX manager 3.1 software (Applied Biosystems). Cq values were computed using the LinregPCR open source software,^58^ and normalized adjusting for plate bias. Corrected Cq that had a s.d. >0.5 were excluded. Relative gene expression levels were determined using the ΔCT method (Biorad real-time PCR application guide, Bio-Rad, #170-9799). Three housekeeping genes (*Actb, Gapdh and Rpl19*) were used to calculate the relative gene expression for all the brain regions-of-interest, except VTA and DS. For VTA, *Actb* was excluded because the negative control displayed amplification, whereas for DS *Gapdh* was excluded because Cq values were found to be significantly different between groups. All the laboratory and preprocessing analyses were performed in a blind manner.

### Corticosterone analyses

Samples were analyzed using the commercial ImmuChem Double Antibody Corticosterone 125^I^ RIA kit for rats and mice (MP Biomedicals, Orangeburg, NY, USA) in accordance with the included protocol, with the exception of the addition of one standard (12.5 ng ml^−1^). All samples were analyzed in duplicate. According to the protocol in the RIA kit, the intra-assay variation was 4.4–10.3% and the inter-assay variation 6.5–7.2%. The corticosterone antiserum showed 100% cross-reactivity with corticosterone, while cross-reactivity to other steroids was 0.34% to deoxycorticosterone, 0.10% to testosterone, 0.05% to cortisol and <0.05% to other tested steroids. Blood corticosterone levels are expressed in terms of ng ml^−1^.

### Statistics

Gene expression data were not normally distributed, except for DS (*P*=0.809), as assessed by Shapiro–Wilk's test, therefore the non-parametric Kruskal–Wallis and Mann–Whitney tests were performed to compare the relative gene expression, corticosterone levels and alcohol intake between three and two experimental groups, respectively. Significance was reported as exact Sig. [2 × (one-tailed Sig.)]. Bivariate correlations were assessed using the non-parametric Spearman test with significance reported as two-tailed significance. To test for correlations between *Maoa* expression in different brain regions and alcohol intake, data of alcohol intake during ADW 6 and 7 were considered. There was homogeneity of variances in gene expression data between groups, as assessed by Levene's non-parametric test for equality of variances (*P*>0.05), therefore the general linear model based on two-way ANOVA, mean of squares type III, was performed to investigate main and interaction effects of MS and alcohol. Whisker box plots were used for illustrative purposes. All statistical analyses were performed using the IBM SPSS software (version 22, IBM, Armonk, NY, USA) and significance was set at *P*⩽0.05.

## Results

*Maoa* expression levels in all brain regions-of-interest are shown for each experimental group in Supplementary Table S2.

### Effect of housing and alcohol consumption on *Maoa* expression in AFR rats

Single housing in adult rats had no effect on *Maoa* expression as indicated by no significant differences between single- and group-housed AFR rats in the VTA, NAc, mPFC, CCx, Amg and DS. Furthermore, there were no significant differences between water- and alcohol-drinking AFR rats, that is, 7 weeks of voluntary drinking had no effect on *Maoa* expression in rats reared in a conventional laboratory setting. The voluntary alcohol consumption in the AFR rats is shown in Supplementary Table S3A.

### Effect of prolonged MS and alcohol consumption on *Maoa* expression in MS rats

**Figure 2:**
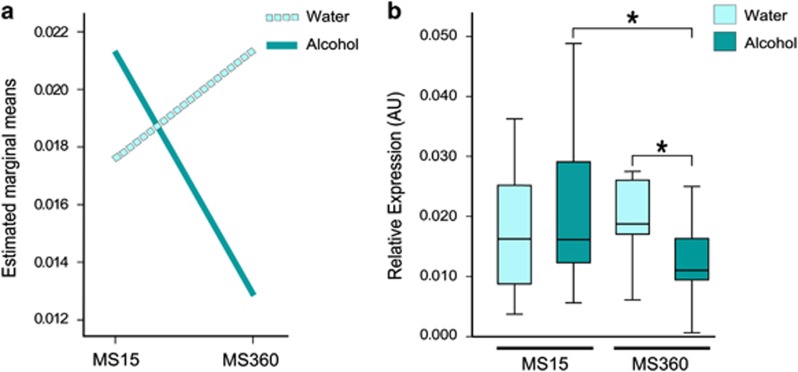
*Maoa* expression in NAc of rats exposed to short or prolonged MS and water or free choice alcohol. (**a**) Interaction effect of stress and alcohol on *Maoa* expression in the NAc in MS15 and MS360 rats (F=3.949; Adj. *R*^2^=0.07; *P*=0.05); (**b**) Lower *Maoa* expression in the NAc of voluntary alcohol-drinking rats exposed to MS360 compared with water-drinking rats (*U*=52; *P=0.05) and MS15 alcohol-drinking rats (*U*=52; *P=0.05). MS15, maternal separation for 15 min; MS360, maternal separation for 360 min; NAc, nucleus accumbens.

**Figure 3:**
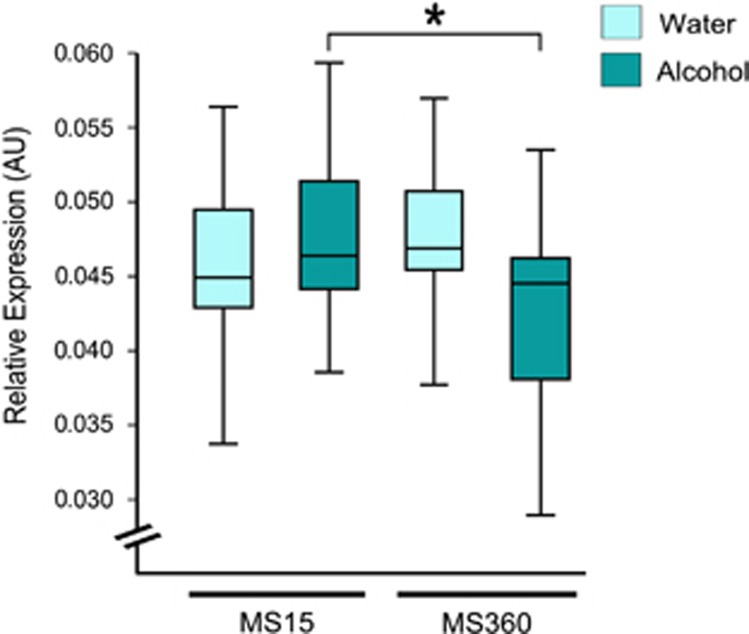
*Maoa* expression in the DS of rats exposed to short or prolonged MS and water or free choice alcohol. Lower *Maoa* expression in the DS of voluntary alcohol-drinking rats exposed to MS360 compared with alcohol-drinking MS15 rats (*U*=55; *P=0.049). DS, dorsal striatum; MS, maternal separation.

The voluntary alcohol consumption in adult MS15 and MS360 rats is shown in Supplementary Table S3A. Alcohol drinking affected *Maoa* expression in the NAc and DS, whereas no differences were found in the VTA, mPFC, CCx and Amg. In the NAc, an interaction effect between MS and alcohol was present (Figure 2a). Alcohol drinking MS360 rats had lower *Maoa* expression compared with water-drinking MS360 rats, whereas no difference was observed between water- and alcohol-drinking MS15 rats (Figure 2b). Furthermore, alcohol-drinking MS360 rats also had lower *Maoa* expression compared with alcohol-drinking MS15 rats (Figure 2b). In the DS, lower *Maoa* expression was found in voluntary alcohol-drinking rats exposed to MS360 compared with MS15 alcohol-drinking rats, whereas no difference was found in comparison to MS360 water-drinking rats (Figure 3). No main or interaction effect of early life stress or alcohol consumption was found in VTA, mPFC, CCx and Amg.

### Correlation between *Maoa* expression and alcohol intake in MS360As rats

As expected,^57, 59^ the majority of the rats that reached a high voluntary consumption (>1.5 g kg^−1^ per 2 h) during the ADW 6 (S16 to S18) and ADW 7 (S19 to S21) were MS360 rats (Supplementary Table S3A, Supplementary Figure S2). To assess correlations between individual drinking behavior and *Maoa* expression in different brain regions, the average alcohol intake for ADW 6 and ADW 7 were considered (see materials and methods). Rats exposed to MS360 were sub-grouped into low (⩽ 1.5 g kg^−1^ per 2 h) and high (> 1.5 g kg^−1^ per 2 h) alcohol-drinking rats according to ADW 6 (Supplementary Table S3B) and ADW 7 (Supplementary Table S3C). According to ADW 6 sub-grouping, no correlation was found between expression in any of the brain region-of-interest and alcohol intake at ADW 6 in MS360 high as well as low alcohol-drinking rats. While according to ADW 7 subgroupings, a strong negative correlation was found between alcohol intake at ADW 7 and expression in DS in MS360 high alcohol-drinking rats (*r*=−0.943, *P*=0.005). In MS360 low alcohol-drinking rats, no correlation was found between expression in any of the brain region-of-interest and alcohol intake at ADW 7. Also, *Maoa* expression was not significantly different in high compared with low alcohol-drinking MS360 rats depending on both ADW 6 and 7, in any of the brain region-of-interest.

### Correlation of *Maoa* expression between brain regions-of-interest in each experimental group

Group-wise correlations were found between *Maoa* expression in different brain regions, mainly between NAc and mPFC (Table 1). In all groups, except the rats exposed to early life stress and alcohol (MS360As), *Maoa* expression in NAc and mPFC was positively correlated. In addition, scattered and group-dependent correlations were also observed between the other regions (Table 1).

### Effect of prolonged MS and alcohol consumption on corticosterone levels in MS rats

**Figure 4:**
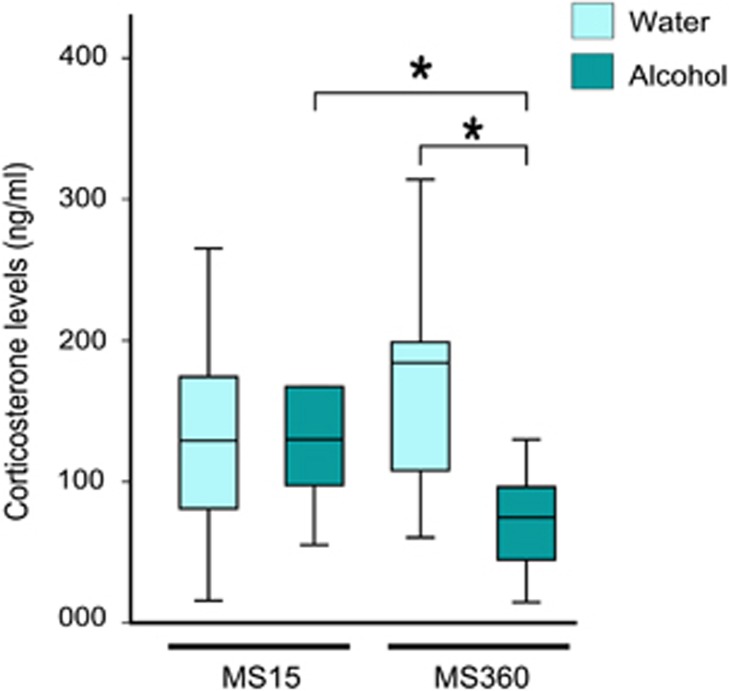
Blood corticosterone levels in MS rats. Lower blood corticosterone levels in MS360 voluntary alcohol-drinking rats compared with alcohol-drinking MS15 rats (*U*=22; *P=0.033), as well as compared with water-drinking MS360 rats (*U*=20; *P=0.003). MS15, maternal separation for 15 min; MS360, maternal separation for 360 min.

Alcohol drinking MS360 rats displayed lower corticosterone levels compared with alcohol-drinking MS15 (*U*=22; *P*=0.033), and water-drinking MS360 rats (*U*=20; *P*=0.003) (Figure 4). While between water- and alcohol-drinking MS15 rats, no significant difference was found in the corticosterone levels, likewise between MS15 and MS360 water-drinking rats.

Group-wise correlations were studied between blood corticosterone levels and *Maoa* expression in different brain regions (VTA, NAc, mPFC, CCx, Amg and DS). No correlation was found in water- or alcohol-drinking MS360 rats, but a positive correlation in Amg in water-drinking MS15 rats (*r*=0.842, *P*=0.002).

## Discussion

### Early life stress, voluntary alcohol consumption and *Maoa* expression

The effect of voluntary episodic alcohol consumption on *Maoa* expression was examined in stress- and reward-related key brain regions in young adults outbred male Wistar rats subjected to early life stress (MS360) or no stress. Lower *Maoa* expression was found in NAc and DS of ethanol drinking MS360 rats. These results are in keeping with human studies where a *MAOA-uVNTR* genotype conferring low transcriptional activity was more common in adolescent males, who had poor family relationship or had been sexually abused or maltreated in childhood, and self-reported higher alcohol consumption.^44, 47^ The present findings suggest a heightened sensitivity of the NAc and DS monoaminergic systems of rats with a background of early life stress to the effects of alcohol, compared with rats without a stressful background.

Monoamines are expressed pre- and post-synaptically during early post-natal development, and have a critical role in modulation of neurodevelopment of functional limbic circuits that allows adaptations to environmental factors.^26^ *MAOA* is indeed strongly expressed during embryonic life in all serotoninergic neurons progressively declining from PND 0 to 10, in adrenergic neurons and post-natally in most transient and permanent dopaminergic neurons.^60^ *Maoa* knock-out mice^61^ and transgenic *Maoa*-deficient mice^62^ are maximally affected during early post-natal life displaying higher levels of monoamines, and stress-related phenotypes including increased aggression in adulthood. Thus, considering the role of *Maoa* in developmental processes shaping adult behavior,^26^ and that early life stress impacted in the present study during a sensitive period of monoaminergic development, it is likely that MS360 rats have a *Maoa*-related sensitivity to alcohol linked to early life stress. It could be hypothesized that ELS would alter *Maoa* expression in other stress-related regions, such as the locus coeruleus and raphe nuclei, of voluntary alcohol-drinking MS360 rats; however, these regions were not available for investigation in the present study.

*Maoa* expression in NAc and mPFC was positively correlated in all groups, except in alcohol-drinking MS360 rats. A straightforward functional interaction exists between mPFC and NAc via dopaminergic neurotransmission in such a way that they are involved with opposite effects on reward mechanisms.^63^ Depletion of dopaminergic activity in mPFC enhances stress as well as reward induced dopamine release in NAc hence acting as some kind of negative feedback, and disruption of this inhibitory control increases the vulnerability to alcohol misuse.^63^ The present correlative pattern points to a disrupted monoaminergic functional interaction of NAc with mPFC following exposure to both early life stress and subsequent consumption of alcohol. Thus, brain region- as well as network-based alterations of MAOA likely underpin early life stress-mediated propensity to alcohol consumption.

Elevated brain *MAOA* expression and blood corticosterone levels are a result of chronic stress and putative risk factors for psychiatric disorders.^64^ Corticosterone levels were higher in water compared with alcohol-drinking MS360 rats, as well as *Maoa* expression in NAc and tendentially in DS, indicating signs of stress. Nevertheless, in rats exposed to early life stress no correlation was found between blood corticosterone and *Maoa* expression, neither in water nor alcohol-drinking rats, in any brain region. Yet, a previous study, employing peri-pubertal stress, reported higher blood corticosterone levels and fronto-cortical *Maoa* expression in stressed adult male rats.^65^ In the present study, after consumption of alcohol, MS360 rats displayed lower corticosterone levels and *Maoa* expression. Hence MS360 rats likely consumed alcohol for stress relieving purpose, which in turn alleviated stress, as illustrated by lower corticosterone and *Maoa* expression in these rats.

### Voluntary alcohol consumption and *Maoa* expression

Lower *Maoa* expression in NAc and DS presumably leads to decreased metabolism and subsequently increased synaptic concentration of monoamines, accompanied by an increased rewarding response to alcohol. Alcohol intake during the 7 weeks of voluntary consumption increased over time in rats exposed to prolonged MS. As expected from earlier studies,^56, 57, 59^ subgroups were observed with some MS360 rats acquiring higher consumption throughout the drinking period. When investigating *Maoa* expression in MS360 rats that acquired a high alcohol intake at ADW 6 and 7, a strong negative correlation was observed between alcohol intake at ADW 7 and *Maoa* expression in DS; whereas no significant difference was found in any of the brain regions-of-interest comparing MS360 rats with high vs low alcohol intake. Similarly, no difference in MAOA activity has been found in whole brain homogenate from alcohol compared with non-alcohol-preferring rats,^66^ in the brainstem, cerebellum, cortex, hippocampus and striatum of rats exposed to alcohol for 90 weeks,^67^ and in the whole brain of rats exposed to both voluntary alcohol intake (1 h per day, 34 weeks) as well as alcohol vapors (5 h per day, 7 weeks).^68^ While upregulation of *Maoa* in CCx of Wistar rats has been reported 3 weeks after termination of long-term exposure to ethanol vapors, NAc was not investigated.^17^ Also, a meta-analysis reported enhanced monoamines levels in forebrain regions of rats after acute alcohol exposure.^12^ In humans, MAOA activity was found to be lower in the hypothalamus and caudate nucleus of chronic alcoholics compared with healthy subjects.^66^ Moreover, low *MAOA* expression in blood and in the core of the NAc associated with high chronic voluntary alcohol consumption in male non-human primates.^38^ It is plausible that methodological differences could explain these discrepant results (for example, alcohol-drinking paradigm and duration).

Previous studies have mainly assessed levels of monoamines (that is, 5-HT, DA and NE) and their major metabolites (that is, 5-hydroxyindoleaceticacid (5-HIAA), homovanillic acid and 3-methoxy-4-hydroxyphenylglycol (MHPG), respectively) in cerebrospinal fluid^48, 69, 70^ or brain tissue,^71, 72^ to investigate alterations in monoamine function and metabolism in the brain of high alcohol-drinking rats or alcoholics. Rats selectively bred for high alcohol drinking were reported having lower levels of 5-HT and 5-HIAA in the hypothalamus, cerebral cortex, DS, NAc, and lower levels of DA and 3,4-dihydroxyphenylacetic acid and homovanillic acid in the NAc and anterior striatum, compared with low alcohol-drinking rats,^72^ likewise in alcohol-preferring compared with non-preferring rats.^73, 74^ Lower rate of DA metabolism and higher DA cerebrospinal fluid levels were observed in rhesus macaques after 12 months of high voluntary alcohol consumption.^38^ In humans, low levels of monoamine metabolites were found in the cerebrospinal fluid of alcoholic patients.^69, 70, 75^ A study of Finnish male criminal alcoholics found lower levels of homovanillic acid and MHPG compared with control but no difference observed in 5-HIAA levels, and a trend of association between the low activity *MAOA*-uVNTR with higher levels of homovanillic acid, 5-HIAA metabolite levels in both alcoholics as well as healthy subjects.^48^ Altogether, these studies provide corroborative evidence of an alcohol-driven downregulation of monoaminergic neurotransmission, though the underlying mechanisms remain unknown.

Rats lack the *MAOA*-uVNTR polymorphism, on the contrary to human and non-human primates. Some, but not all,^48, 49^ human studies report an association between the low transcriptional activity allele of *MAOA*-uVNTR and higher risk of AUD.^42, 43^ In addition, in non-human primates the high transcriptional activity allele of rh*MAOA*-uVNTR was found to be associated with high alcohol consumption,^51^ or no association was found.^38^ Discrepancies in genetic association studies possibly reflect the contradictory *in vitro*,^34, 35^ relative to *in vivo,*^36, 37, 38^ findings about the functionality of this polymorphism. In addition, they make room to speculate on early-in-time susceptibility, which in turn might be smoothed over time by epigenetic mechanisms in a favorable or disadvantageous manner. In fact, low blood *MAOA* expression, but not rh*MAOA*-uVNTR genotype, predicts high voluntary alcohol consumption in rhesus macaques.^38^ Taken together with the present findings, there is suggestive evidence that both the transient decrease in *Maoa* expression following alcohol exposure as well as low levels of MAOA throughout life time (presumably due to genetic variation in the *MAOA)* would predict risky alcohol consumption behavior in adulthood.

### Methodological strengths and limitations

Wistar pups were subjected to early life stress in a rodent model showing good face and construct validity in studies of early life impact on adult voluntary alcohol consumption.^55, 56^ Prolonged maternal separation (MS360) every day for the first 3 PNWs simulates early life stress through disturbed social interactions, and is a risk environment for high adult alcohol consumption, whereas MS15 is associated with low alcohol intake.^56, 76^ Besides providing a protective environment, MS15 serves as control for MS360 since rats in both groups are exposed to the same handling procedures.

In adulthood, rats were single-housed and given a two-bottle free choice between water and alcohol with intermittent access to alcohol (3 consecutive days followed by 4 days of no alcohol with only access to water) to mimic episodic alcohol drinking in humans,^77^ for a limited period (2 h),^78, 79^ for 7 successive weeks. Studies suggest that intermittent and limited access to alcohol induces higher voluntary alcohol intake compared with continuous access for 24 h^80, 81, 82, 83^ and have the advantage of producing neurochemical adaptations that are relevant to AUD.^84^

The MS360 rats were sub-grouped into high (>1.5 g kg^−1^ per 2 h) and low (⩽1.5 g kg^−1^ per 2 h) alcohol-drinking groups based on previous knowledge of blood alcohol concentration (BAC) depending on amount of alcohol consumed. For example, rats at 10 weeks of age had BAC≈70 mg dl^−1^ after 1.5 mg kg^−1^ alcohol intake, as similarly shown in adult rats having a BAC≈80 mg dl^−1^ 2 h after exposure to 2 g kg^−1^.^85^ Nevertheless, the high alcohol consumption level of 1.5 g kg^−1^ per 2 h is relative to the Wistar outbred rats used in the present study. Though the alcohol consumption is low compared with rats in the addictive state or genetically alcohol-preferring rats, the purpose of the present study was to investigate early neurobiological signatures related to episodic voluntary alcohol intake in non-addicted and non-preferring rats.

As single housing in the home cage is required in voluntary drinking paradigms, the effect of single housing on *Maoa* expression in adult AFR rats, that is, rats subjected to conventional rearing conditions, was studied in the present study. Single housing did not affect *Maoa* expression when compared with group housing, indicating no confounding effects due to housing conditions. Furthermore, voluntary alcohol drinking did not affect *Maoa* expression in the AFR rats in comparison to water-drinking rats hence suggesting that voluntary alcohol consumption does not affect *Maoa* expression in normal or non-stressful conditions (animal facility rearing, in this case).

Only male rats were herein investigated calling for studies of females. Sex differences have been implicated in human studies on *MAOA*-uVNTR genotype, and its interaction with early life environment, as well as in rodents regarding the impact of maternal separation on voluntary alcohol intake.^76, 86^ Moreover, ample evidence exists of influences of the gonadal hormones on the monoaminergic mesocorticolimbic circuit functioning and the response to stressors and vulnerability to psychiatric illness.^87, 88, 89^

The present study tested a previously described definite hypothesis, and no correction for multiple testing was applied. Restrictions, such as the Bonferroni correction, are concerned with a general null hypothesis (H_0_) and are employed to reduce type I errors, as well as reduce the statistical power to reject a false null hypothesis rendering truly important differences as non-significant since findings depend on a number of other tests performed that in turn inflate the likelihood of accepting type II errors.^90, 91^

## Conclusions

Knowledge of the effects of voluntary alcohol drinking on *Maoa* expression in brain regions related to reward and addiction is limited. The present results show that voluntary drinking in adulthood was associated with low *Maoa* expression in the NAc and DS but only in rats previously exposed to early life stress. No differences were seen in water-drinking control rats exposed to stress or no stress, indicating that voluntary alcohol consumption is necessary to reveal a monoaminergic dysfunction in animals exposed to early life adverse conditions. These findings expand current knowledge on the molecular basis of the previously reported associations between early life stress, *Maoa* and susceptibility to excessive alcohol consumption and AUD. Further studies are needed to investigate mechanistic signatures behind *MAOA*-driven susceptibility to AUD. Efforts should be convened to investigate the mesocorticolimbic monoaminergic system during early life and at an early stage of alcohol-drinking behavior.

## Figures and Tables

**Table 1:** Group-wise correlations of *Maoa* expression in different brain regions-of-interest^a^

*Group*	*Regions*	n	*Correlation*	*VTA*	*mPFC*	*CCx*	*DS*
AFR Wg	NAc	9	r		0.733		
			*p*	—	0.025	—	—
AFR Ws	NAc	9	r		0.717		
			*p*	—	0.030	—	—
AFR As	NAc	11	r		0.627		
			*p*	—	0.039	—	—
	mPFC	11	r				−0.755
			*p*	—	—	—	0.007
MS15 Ws	NAc	10	r		0.745		
			*p*	—	0.013	—	—
	mPFC	10	r	0.709			
			*p*	0.022	—	—	—
	Amg	10	r	−0.697			
			*p*	0.025	—	—	—
MS360 Ws	NAc	10	r	0.673	0.636		
			*p*	0.033	0.048	—	—
	mPFC	10	r	0.855			
			*p*	0.002	—	—	—
	CCx	10	r				−0.648
			*p*	—	—	—	0.043
MS15 As							
	VTA	10	r			0.818	
			*p*	—	—	0.004	—
	NAc	10	r		0.661		
			*p*	—	0.038	—	—
MS360 As							
		19	r				
			*p*	—	—	—	—

Abbreviations: A, alcohol-drinking rats; AFR, animal facility reared; Amg, amygdala; CCx, cingulate cortex; DS, dorsal striatum; g, group housed; mPFC, medial prefrontal cortex; MS15, maternal separation for 15 min; MS360, maternal separation for 360 min; *n*, total number of rats; NAc, nucleus accumbens; *P*, level of statistical significance; *r*, Spearman's correlation coefficient; s, single-housed; VTA, ventral tegmental area; W, water-drinking rats.

a Only moderate and high correlations (*r*⩾0.5) are reported.

## Supplementary Material

Supplementary Informations
