# Supplementary material for: Effect of voluntary alcohol consumption on Maoa expression in the mesocorticolimbic brain of adult male rats previously exposed to prolonged maternal separation
Source: Transl Psychiatry. 2015 Dec 8;5(12):e690–. doi: 10.1038/tp.2015.186 (PMC5068586; doi:10.1038/tp.2015.186)
Supplement: Supplementary Informations [file tp2015186x1.docx]

**Table S1**

Gene specific primers and relative annealing temperature

| **Gene** | **Description** | **Primers** | | **T (^o^C)** |
| --- | --- | --- | --- | --- |
| *Actb* | Actin beta | Forward | 5' CAC TGC CGC ATC CTC TTC CT 3' | 60.0 |
|  |  | Reverse | 5' AAC CGC TCA TTG CCG ATA GTG 3' |  |
| *Gapdh* | Glyceraldehyde-3-phosphate dehydro genase | Forward | 5' ACA TGC CGC CTG GAG AAA CCT 3' | 60.0 |
|  |  | Reverse | 5' GCC CAG GAT GCC CTT TAG TGG 3' |  |
| *Maoa* | Monoamine oxidase A | Forward | 5' CTA TGT GGG ACC AAC CCA GA 3' | 64.5 |
|  |  | Reverse | 5' AAT ATG CCA AGG GGT TCC AC 3' |  |
| *Rpl19* | Ribosomal protein L19 | Forward | 5' TCG CCA ATG CCA ACT CTC GTC 3' | 62.0 |
|  |  | Reverse | 5' AGC CCG GGA ATG GAC AGT CAC 3' |  |

**Table S2**

Median group-wise relative gene expression of *Maoa* in the brain regions of interest.

| **Group** | **AFR Wg**  **(Min-Max)** | **AFR Ws**  **(Min-Max)** | **AFR Es**  **(Min-Max)** | **MS15 Ws**  **(Min-Max)** | **MS360 Ws**  **(Min-Max)** | **MS15 Es**  **(Min-Max)** | **MS360 Es**  **(Min-Max)** |
| --- | --- | --- | --- | --- | --- | --- | --- |
|  | **(n = 9)** | **(n = 9)** | **(n = 11)** | **(n = 10)** | **(n = 10)** | **(n = 10)** | **(n = 20)** |
| **VTA** | 0.055^b^  (0.042-0.089) | 0.050  (0.030-0.106) | 0.050  (0.029-0.102) | 0.075  (0.022-0.137) | 0.045  (0.029-0.083) | 0.061  (0.024-0.096) | 0.065 ^c^  (0.022-0.104) |
| **NAc** | 0.018  (0.008-0.043) | 0.017  (0.007-0.073) | 0.017  (0.006-0.050) | 0.016  (0.004-0.036) | 0.019  (0.006-0.055) | 0.016  (0.006-0.049) | 0.011^a^  (0.001-0.025) |
| **mPFC** | 0.012  (0.004-0.071) | 0.009  (0.004-0.025) | 0.011  (0.005-0.036) | 0.011  (0.004-0.025) | 0.010  (0.005-0.023) | 0.010  (0.006-0.023) | 0.013  (0.005-0.023) |
| **CCx** | 0.023  (0.020-0.090) | 0.026  (0.021-0.095) | 0.028  (0.019-0.103) | 0.026  (0,020-0.072) | 0.025  (0.021-0.097) | 0.028  (0.019-0.127) | 0.071  (0.020-0.131) |
| **Amg** | 0.025^a^  (0.021-0.027) | 0.023  (0.021-0.030) | 0.022  (0.019-0.028) | 0.023  (0.018-0.033) | 0.024  (0.020-0.031) | 0.024  (0.019-0.028) | 0.022  (0.020-0.031) |
| **DS** | 0.049  (0.044-0.059) | 0.046  (0.037-0.053) | 0.043  (0.031-0.055) | 0.045  (0.034-0.056) | 0.046  (0.039-0.059) | 0.047  (0.038-0.057) | 0.045  (0.029-0.054) |

AFR: Animal facility reared; Amg: Amygdala; CCx: Cingulate cortex; DS: Dorsal striatum; E: ethanol drinking rats; g: group housed; mPFC: Medial prefrontal cortex; MS15: Maternal separation for 15 min; MS360: Maternal separation for 360 min; n: total number of rats; NAc: Nucleus Accumbens; s: single housed; SD: standard deviation; VTA: ventral tegmental area; W: water drinking rats. ^a^ _=_ one rat excluded due to technical problems; ^b^ _=_ two rats excluded due to technical problems; ^c^ _=_ three rats excluded due to technical problems

**Table S3**

1. Group-wise voluntary alcohol intake (median) over seven weeks *.

| **Group** | **n** | **ADW 1**  **(5% / 24`h)**  **(Min-Max)** | **ADW 2**  **(5% / 2 h)**  **(Min-Max)** | **ADW 3**  **(20% / 2 h)**  **(Min-Max)** | **ADW 4**  **(20% / 2 h)**  **(Min-Max)** | **ADW 5**  **(20% / 2 h)**  **(Min-Max)** | **ADW 6**  **(20% / 2 h)**  **(Min-Max)** | **ADW 7#**  **(20% / 2 h)**  **(Min-Max)** |
| --- | --- | --- | --- | --- | --- | --- | --- | --- |
| **AFRAs** | 11 | 1.61  (0.29-4.16) | 0.40  (0.18-0.75) | 1.10  (0.50-2.11) | 1.21  (0.59-1.43) | 1.37  (0.77-2.08) | 1.18  (0.62-1.66) | 1.07  (0.71-1.75) |
| **MS15As** | 10 | 1.89  (0.87-3.40) | 0.67  (0.32-0.99) | 1.12  (0.75-1.61) | 1.49  (0.89-2.13) | 1.23  (1.05-1.74) | 1.32  (0.39-1.77) | 1.32  (0.50-1.84) |
| **MS360As** | 20 | 1.53  (0.21-3.28) | 0.57  (0.09-1.14) | 1.20  (0.29-1.98) | 1.11  (0.56-2.15) | 1.28  (0.38-2.52) | 1.32  (0.60-2.05) | 1.22  (0.64-2.52) |

B. Trajectory of voluntary alcohol intake (median) of low and high alcohol drinking MS360 rats according to ADW 6*.

| **MS360As subgroups** | **n** | **ADW 1**  **(5% / 24 h)**  **(Min-Max)** | **ADW 2**  **(5% / 2 h)**  **(Min-Max)** | **ADW 3**  **(20% / 2 h)**  **(Min-Max)** | **ADW 4**  **(20% / 2 h)**  **(Min-Max)** | **ADW 5**  **(20% / 2 h)**  **(Min-Max)** | **ADW 6**  **(20% / 2 h)**  **(Min-Max)** | **ADW 7#**  **(20% / 2 h)**  **(Min-Max)** |
| --- | --- | --- | --- | --- | --- | --- | --- | --- |
| **Low**  ≤ 1.5 g/kg/2h | 13 | 1.54  (0.21-3.28) | 0.57  (0.09-0.80) | 1.15  (0.29-1.85) | 1.03  (0.56-1.45) | 1.20  (0.71-2.52) | 0.99  (0.60-1.41) | 0.93  (0.64-1.33) |
| **High**  >1.5 g/kg/2h | 7 | 1.13  (0.22-2.74) | 0.65  (0.25-1.14) | 1.24  (0.94-1.98) | 1.61  (0.80-2.15) | 1.51  (0.38-1.90) | 1.72^a^  (1.65-2.05) | 1.98  (1.46-2.52) |

C. Trajectory of voluntary alcohol intake (median) of low and high alcohol drinking MS360 rats according to ADW 7*.

| **MS360As subgroups** | **n** | **ADW 1**  **(5% / 24 h)**  **(Min-Max)** | **ADW 2**  **(5% / 2 h)**  **(Min-Max)** | **ADW 3**  **(20% / 2 h)**  **(Min-Max)** | **ADW 4**  **(20% / 2 h)**  **(Min-Max)** | **ADW 5**  **(20% / 2 h)**  **(Min-Max)** | **ADW 6**  **(20% / 2 h)**  **(Min-Max)** | **ADW 7#**  **(20% / 2 h)**  **(Min-Max)** |
| --- | --- | --- | --- | --- | --- | --- | --- | --- |
| **Low**  ≤ 1.5 g/kg/2h | 14 | 1.64  (0.21-3.28) | 0.57  (0.09-0.80) | 1.14  (0.29-1.85) | 1.02  (0.56-1.45) | 1.23  (0.71-2.52) | 1.01  (0.60-1.86) | 0.97  (0.64-1.46) |
| **High**  >1.5 g/kg/2h | 6 | 0.96  (0.22-2.74) | 0.46  (0.25-1.14) | 1.32  (0.94-1.98) | 1.64  (0.80-2.15) | 1.42  (0.38-1.77) | 1.70  (1.65-2.05) | 1.98 ^a^  (1.62-2.52) |

AFR: Animal facility reared; A: alcohol drinking rats; ADW: Alcohol drinking week; h:hour; MS15: Maternal separation for 15min; MS360: Maternal separation for 360min; n: total number of rats; s: single housed.

*: values are expressed as median calculated for average alcohol intake of each rat for three sessions for each respective ADW.

a: Alcohol intake was significantly higher in high alcohol drinkers compared to low (Mann Whitney U = 0.00, *p* ≤0.001).

**Figure S1**

1. Gel image of the qPCR product specificity in different brain regions of interest.


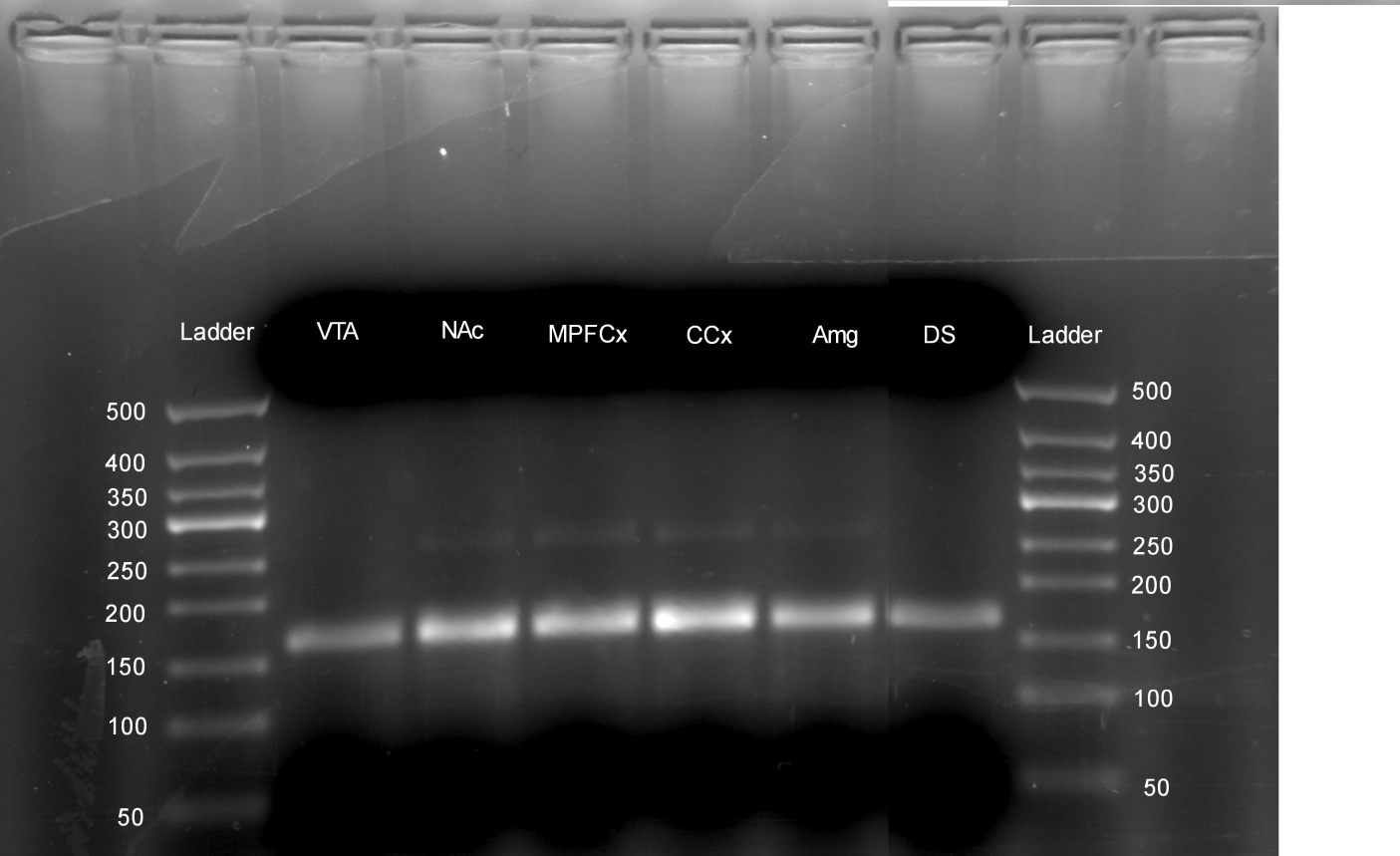


Amg: Amygdala; CCx: Cingulate cortex; mPFC: Medial prefrontal cortex; NAc: Nucleus Accumbens; DS: Dorsal striatum; VTA: ventral tegmental area.

1. Gel image showing RNA integrity for a randomly selected 10% of samples in each brain region.
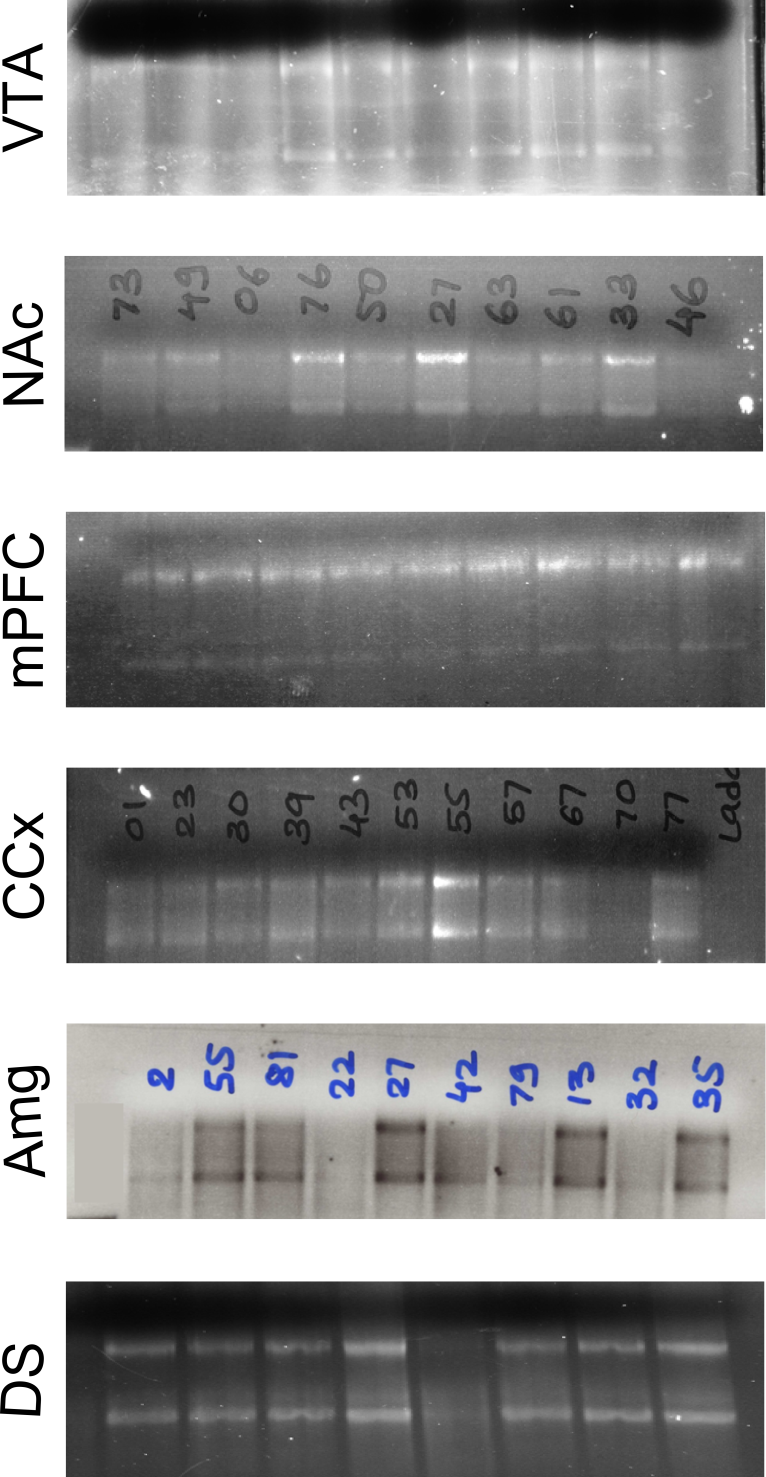


In each region, Out of the two bands, the one at the first [^1^](#_ENREF_1) band represents 28S and the second [^2^](#_ENREF_2) band represents 18S subunit of RNA. Amg: Amygdala; CCx: Cingulate cortex; mPFC: Medial prefrontal cortex; NAc: Nucleus Accumbens; DS: Dorsal striatum; VTA: ventral tegmental area.

**Figure S2**

Trajectory of voluntary alcohol intake of each group over seven weeks


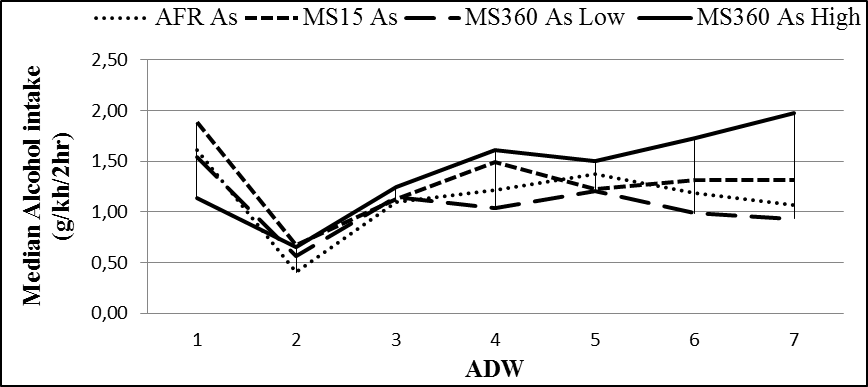


A: alcohol drinking rats; AFR: Animal facility reared; ADW: Alcohol drinking week; h: hour; MS15: Maternal separation for 15min; MS360: Maternal separation for 360min; s: single housing.
